# Preventing the risk of iatrogenic harm when assessing and diagnosing functional neurological disorders and other functional somatic symptoms

**DOI:** 10.1192/j.eurpsy.2024.1778

**Published:** 2024-09-25

**Authors:** Giovanni Stanghellini, George Ikkos, Cecilia Maria Esposito

**Affiliations:** 1 Department of Health Sciences, University of Florence, Florence, Italy; 2 Centro de Estudios de Fenomenologìa y Psiquiatrìa, ‘Diego Portales’ University, Santiago, Chile; 3 Royal National Orthopaedic Hospital, Stanmore, UK; 4 Department of Neurosciences and Mental Health, IRCCS Fondazione Ca’ Granda Ospedale Maggiore Policlinico, Milan, Italy

**Keywords:** Functional neurological symptoms, functional somatic symptoms, hysteria, iatrogenic harm

## Abstract

The present commentary raises some concerns about the risk of iatrogenic harm arising out of the diagnosis of functional neurologic and somatic disorders. These concerns are supported by evidence from the history of hysteria and findings from contemporary brain imaging. We discuss their implications for practice.

Functional neurological symptoms (FNSs) and functional somatic symptoms (FSSs) including, for example, fibromyalgia and unexplained medical symptoms are clinical presentations that tread the border between the psychic and the somatic. In general, they come much more often to the attention of the general practitioner or neurologist than the psychiatrist. Terminology is varied and can be confusing but Creed et al. [1] report that medically unexplained symptoms are the presenting problem in up to 35–53% of new patients at specialist medical clinics and FSS have been found to be present in up to a third of healthcare consultations both in primary care and in specialist practice [2]. With respect to functional neurological disorders (FNDs), FSS, and FND/psychiatric comorbidity, Carle-Toulemonde et al. [3] found that fatigue is the most common symptom reported in FND (from 47 to 93%). Psychopathological disorders are reported in 40 to 100% FND patients, anxiety disorders being the most frequent, followed by mood disorders and neurodevelopmental disorders. Stress factors such as childhood trauma (mainly emotional neglect and physical abuse) have also been identified in up to 75% of FND patients, along with maladaptive coping strategies.

Recently, neurologists have paid greater attention to these clinical conditions, leading to important reflections. For example, Stone et al. [4] propose to overcome the conceptual mind–body gap by understanding FND as a “predictive brain” disorder. Changes observed with neuroimaging techniques in the network involved in sense of agency “–the parts of the brain that let you know that it is ‘you’ who made a movement–”, as they argue, may explain why it would be difficult for FND patients to acquire awareness of their own bodily state and movements and thus adjust future action in the light of accurate perceptual feedback.

We applaud the endeavor to promote the constructive engagement of neurologists in the care of patients with FND. It is a major advance in clinical practice. However, we also have some concern that a perverse effect of this genuine progress may be to lead some psychiatrists to conclude that, for practical purposes, FND is a brain or neurological disorder. This may lead to losing out on useful insights generated by the rich history of different approaches. Therefore, we stress that each patient presenting with FNS and FSS requires full assessment of all relevant clinical phenomena, not only neurological ones, but also psychopathological and psychological factors. Also, we must consider not only the neurological and psycho(patho)logical correlates of functional disorders, but also the sociocultural roots that often underly them.

Psychiatric disorders, including neuropsychiatric syndromes, are not a-temporal entities but are influenced by changes in society and the medical paradigm. Changes in medical knowledge can produce changes in the presentation of clinical conditions as well as their medical formulation and treatment. Arguably, FND, FSS, etc., are (at least in part) modern labels for “hysteria” – a set of clinical phenomena that have historically been greatly influenced by the social context. The term “hysteria” has justly been criticized for its misogynistic provenance and associated discriminatory practice, but this does not mean we cannot learn from its history in a way that the total abolition of reference to the term would make impossible [5]. Here, we focus on some medical and social dynamics that may contribute to the onset, maintenance, and adverse prognosis of these varied and overlapping clinical presentations, if not appropriately addressed. For the avoidance of doubt: in advocating our thesis below, we do not suggest that every patient who presents with functional or medically unexplained symptoms conforms to our analysis. Only a significant number does, and this is across the various contemporary diagnoses of FND, FSS, etc.

Hysterical symptoms are known to be highly influenced by the doctor–patient relationship. Didi-Huberman [6] has described how the “modern” delineation of hysteria, dating back to Charcot during the 19th century, was strongly affected by a climate of seductiveness between doctor and patient. To get doctors’ attention, this apparently led the latter to “mimic” hysteria in line with then current medical formulations of the condition. The need for recognition by others, which represents an evolutionarily acquired profound human need [7], becomes the only way for people in the grip of hysterical dynamics to feel validated in their existence. Medical malpractice, when it has often ridiculed or overseen these presentations, has then served to exacerbate this need.

For those with a magmatic and inconstant perception of their own body from within (i.e., coenesthetically), the optical imprint of the other on themselves takes on particular importance: “*Videor ergo sum* - I am seen therefore I am” [5]. The core of hysteria has in fact been described as a “*manque d’être*” (‘lack of being’). The attempt to compensate for this lack gives rise to the need to adapt to models and identifications proposed from outside [8]. While we agree that patients presenting with these symptoms are not malingering, fabricating, or pretending, or lying, we would argue that their utterances and behavior have a performative function, that of capturing the gaze of the clinician. We refer here to the interested, not the dismissive clinician!

It is not surprising that the medical gaze, with its promise not only of recognition but also of diagnostic definition, has over time been one of the privileged targets of hysterical capture. The medical gaze serves the search for identity and diagnosis and represents a “device of subjectivation” [9] for these patients. Some, medical doctors, in turn, respond with great attentiveness, as such presentations literally embody a fundamental question that has fascinated medicine since its beginnings – the mysterious relationship between soma and psyche. By embodying this mystery, people with hysterical symptoms catch the medical eye and gain much needed attention and recognition. Furthermore, the anthropological disproportion that sees the optical dimension prevail over the coenesthetic dimension is in accordance with the “*Zeitgeist”* of today [10]. Biomedicine, with its array of imaging techniques, is one of those “machines to be seen and to be talked about” that philosophers [11] refer to. These help to produce a specific kind of subjectivity through a particular kind of process of subjectivation and this mutation has its epicenter in the “society of the image” [10]. To this general social phenomenon, biomedicine adds its contribution by making the body itself the object of ocular knowledge [9].

From these considerations, a question arises: since hysterical dynamics, including their contemporary declinations as FNS and FSS, are nourished by medical attention and by the visibility so obtained, are we always truly serving the patient’s interest by making these diagnoses or are we reinforcing an intrinsic tendency of their psychopathological functioning? Far from helping clinical discernment, may the diagnosis of FNS and FSS be contributing to iatrogenic harm sometimes?

People who, after a long periplus, finally receive a diagnosis of FNS or FSS are often happy to be recognized as persons suffering from a disease included in medical nosography. Perhaps, sometimes, their aim is to have a diagnosis and medical support, that is, to be investigated as sick without being subjected to the stigma related to a diagnosis of mental disorder – rather than to recover from their symptoms. In addition to this, they may not just be looking for a medical diagnosis and recognition, but also for an *identity.* To be more precise: they may be looking for a diagnosis to identify with. That’s why these diagnoses can contribute to the long-term maintenance of symptoms, rather than their amelioration. Importantly, it will be noted that recent findings on brain imaging [4] are highly consistent with our observations about coenesthetic hypofunction in patients surviving with hysterical dynamics, strengthening our sociocultural approach. Of course, brain imaging cannot aspire to capture adequately the dynamics relating to social and medical history and their impact on clinical consultations in primary, neurological, psychiatric, or other care.

In conclusion, it is important that those who care for patients with functional neurological and somatic symptoms do not stop at diagnosis – which it should be noted, is now a positive diagnosis, not one by exclusion [4]. If we are not to miss any facet of the patients’ experience, it is essential to develop an appropriately rich shared perspective between psychiatrists, neurologists, and GPs. Unless we do so, we will fail to do full justice to the nature of FNS and FSS and the associated distress.
